# Scalable VO_2_-metal metasurface enabling adaptive and frequency-selective infrared switching

**DOI:** 10.1515/nanoph-2025-0074

**Published:** 2025-05-22

**Authors:** Haoxuan Xun, Hang Wei, Jian Chen, Rui Wang, Huan Guan, Hongyu Zhu, Shuliang Dou, Jinxin Gu, Yunbin He, Xiaofeng Li, Yaohui Zhan

**Affiliations:** School of Optoelectronic Science and Engineering & Key Lab of Advanced Optical Manufacturing Technologies of Jiangsu Province & Key Lab of Modern Optical Technologies of Education Ministry of China, 12582 Soochow University, Suzhou 215006, China; School of Microelectronics, Wuhan Textile University, Wuhan 430200, China; Center for Composite Materials and Structure, Harbin Institute of Technology, Harbin 150001, China; Suzhou Laboratory, Suzhou 215123, China

**Keywords:** vanadium dioxide, infrared switch, metasurface, frequency-selective, scalable

## Abstract

Infrared windows-enabled infrared inspection has significant applications in both civilian and military domains. However, the static and indiscriminate transparency across the visible to infrared regions renders them vulnerable to potential laser damage. In this study, we construct a dynamic infrared optical switch based on a vanadium dioxide (VO_2_)–Al metasurface structure. The phase transition of VO_2_ enables dynamic transmittance switching, while the Al metasurface confines this capability to specific wavelengths to mitigate unwanted interferences. The infrared switch transmits light within the 8–14 μm range only in its “ON” state, reflecting other wavelengths across the visible to infrared spectrum. The underlying physics is attributed to plasmon-induced extraordinary optical transmission combined with the reconfigurable metatronic properties of the VO_2_ layer. Moreover, while maintaining excellent optical performance, we utilize full-wave simulations to enlarge the feature sizes of the Al metasurface structure to meet the demands for large-area fabrication. This advancement paves the way for practical applications of infrared switches, highlighting significant research implications for intelligent infrared windows and enhancing our understanding of light–matter interactions in reconfigurable photothermal devices.

## 1 Introduction

Infrared windows facilitate the transmission of infrared radiation, enabling effective infrared inspection via thermal cameras for both civilian and military purposes, such as satellite remote sensing, missile guidance, non-invasive surgical monitoring, industrial surveillance and so on [1], [2], [3], [4], [5]. However, traditional infrared windows exhibit static and indiscriminate transparency across a broad spectrum, from visible to mid-infrared wavelengths, which makes them vulnerable to potential laser damage or interference. To address this problem, there is a growing need for dynamic and spectrally selective optical switches that can be integrated into infrared windows [6], [7], [8], [9], [10], [11]. Vanadium dioxide (VO_2_) undergoes a reversible phase transition from dielectric VO_2_(M) phase to metallic VO_2_(R) phase at approximately 68 °C. During the phase transition, conductivity of VO_2_ increases by 3–4 orders of magnitude, which leads to drastic changes in infrared behaviors from transparent to reflective [4], [5], [12], [13], [14], [15], [16], [17]. It makes VO_2_ an ideal candidate for creating self-adaptive optical switches that can dynamically modulate their optical characteristics in response to thermal stimuli.

VO_2_-based infrared switches are primarily classified into two types: emissivity modulation [18], [19], [20], [21], [22], [23], [24] and transmittance modulation [5], [25]. Emissivity modulation functions by switching between low and high emissivity, typically achieved through multilayer cavities or metasurfaces [22], [23], [24], [26] to induce electromagnetic resonance in the metallic VO_2_ state. However, the presence of metal reflectors limits the potential for transmittance modulation, hindering applications that require infrared windows [27], [28], [29], [30]. As for the transmittance modulation type, the weak wavelength-selective optical behaviors of VO_2_ will diminish the effectiveness of VO_2_ films when applied to infrared windows. To mitigate it, frequency selective surfaces (FSS), typically consisting of photonic crystal stacks and metallic nanostructures, are often placed above VO_2_ to facilitate wavelength-selective transmittance modulation.

Recently, King et al. [5] demonstrated an electrical heating controlled VO_2_–metal metasurface which exhibited great transmittance tunability between 3 and 5 μm. The fabricated metasurface had dimensions of 400 µm × 400 µm with a smallest feature of ∼200 nm and the switching are triggered by varying an electrical current. Similarly, Wan et al. [31] fabricate a prototype reflective limiter comprising of an Au cross-slot metasurface with an aperture width of ∼0.17 μm, achieving a high open-state transmittance (∼0.7) and low limiting-state transmittance (∼0.01). Despite these advancements, the small structural sizes of FSS typically restrict sample dimensions to less than 1 mm, which poses challenges for large-scale manufacturing and practical applications. Consequently, there is a pressing need for an infrared switch that combines dynamic, wavelength-selective transmittance modulation with larger microstructures suitable for scalable production. Moreover, while research efforts on infrared switches have primarily concentrated on the mid-infrared regime, their synchronous switching characteristics and dynamic modulation capabilities across the visible and near-infrared regions remain unexplored. Developing a comprehensive understanding of these properties over a broader spectral range is of paramount importance for enabling strict applications involving tunable high-power lasers.

In this work, we propose a dynamic and spectrally selective infrared switch integrated into the infrared window, designed to only transmit mid-infrared light in the normally open state while suppressing other wavelengths, particularly in the visible and near-infrared ranges dominated by high-power lasers. Through comprehensive full-wave simulations, we engineered the optical switch with a highly enlarged microscale minimum feature size (0.86 μm) for large-scale fabrication. Experimental validation of the dynamic spectral modulation of the adaptive switch under heating and cooling processes is conducted across the ultraviolet to mid-infrared spectrum. The underlying physics of spectral switching is investigated through microscopic electromagnetic field analysis and equivalent electrical circuitry, revealing its dependence on plasmon-induced extraordinary optical transmission and the reconfigurable metatronic properties of the phase-change layer. This investigation presents a promising prototype for mid-infrared switches boasting excellent self-adaptiveness, spectral selectivity, and feasibility for large-scale production, representing a significant advancement toward practical intelligent infrared windows and enhancing our understanding of light–matter interactions in reconfigurable photothermal devices.

## 2 Results and discussions

**Figure 1:**
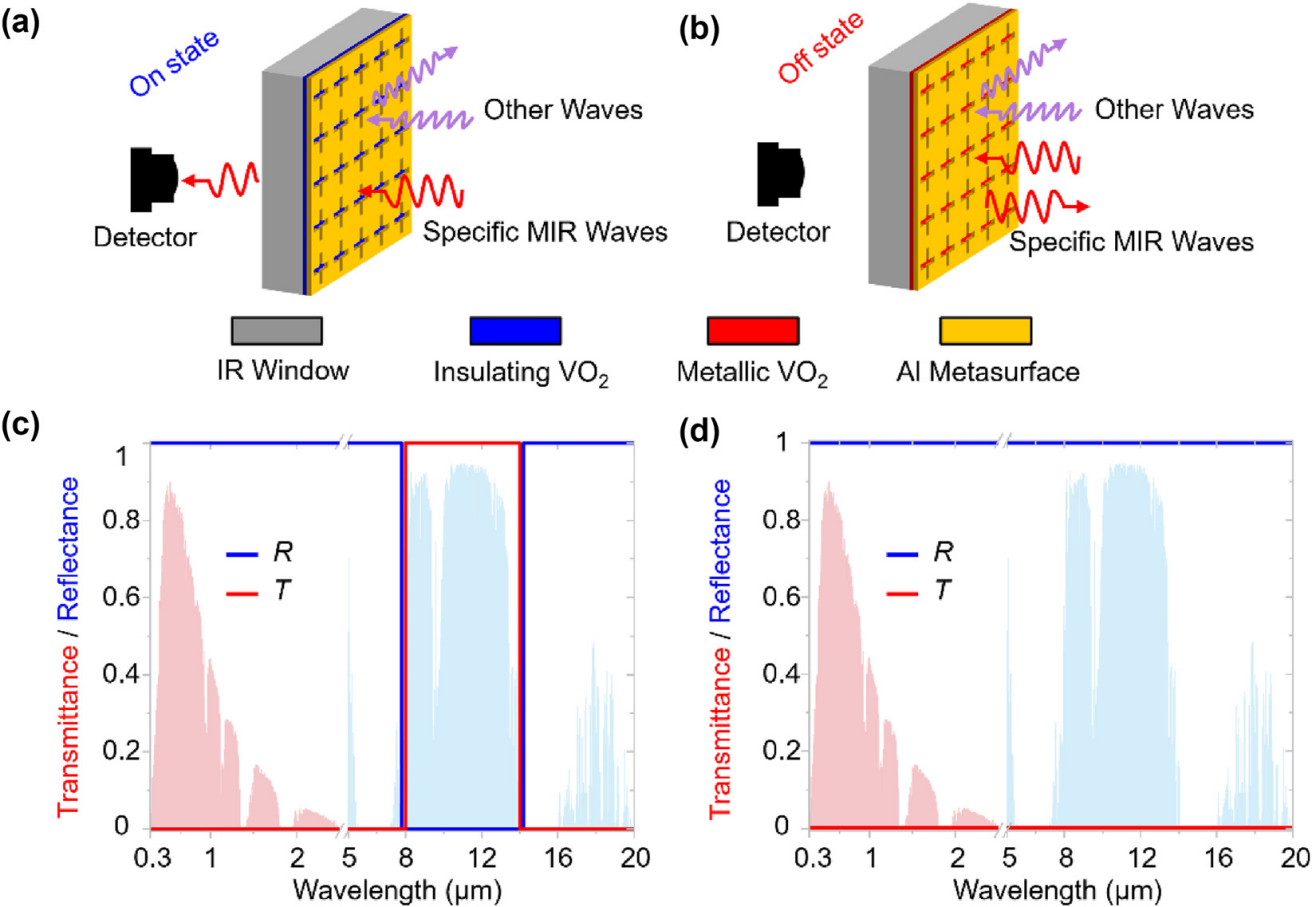
Design purpose and physical concept. (a) ON state and (b) OFF state situations for the infrared detector behind the infrared window that is integrated with the adaptive switch. In the ON state situation, the infrared window is desired to transmit only the mid-infrared light to the thermal imager for signal detection; the other undesired light is expected to be blocked due to potential interferences and threats. Once the external stimulus (e.g., laser irradiation) is imposed, the infrared window would close all the optical channels and turn into the OFF state, making use of the thermo-induced IMT of the phase-change layer. (c) and (d) Ideal transmittance (red line)/reflectance (blue line) spectra for the situations shown in (a) and (b), respectively.

Figure 1a and b sketches the fundamental principle of our device. The device comprises an infrared-transparent CaF_2_ substrate, a phase-change VO_2_ layer, and an Al metasurface layer. Here, we adopt the tunable permittivity from the phase transition of VO_2_ to generate the high transmittance contrast between the ON and OFF states. Then, we design and construct the Al metasurface to enable the frequency-selective switching capacity. Besides, by further optimization, we enlarge the minimum feature size of Al metasurface to micrometers which are 10 times larger than existing reports, which is critical for wafer-scale production. The ideal transmittance and reflectance spectra in the ON and OFF states are given in Figure 1c and d, respectively. When our device switches from the ON state to the OFF state, the transmittance in a specific narrow band (Here we choose 8–14 μm as an example to design the device and optimize the structure) will decrease from 1 to 0, while the reflectance simultaneously rises from 0 to 1. In other wavelengths, the reflectance keeps 1 to avoid light pollution and potential interference with photodetectors.

**Figure 2:**
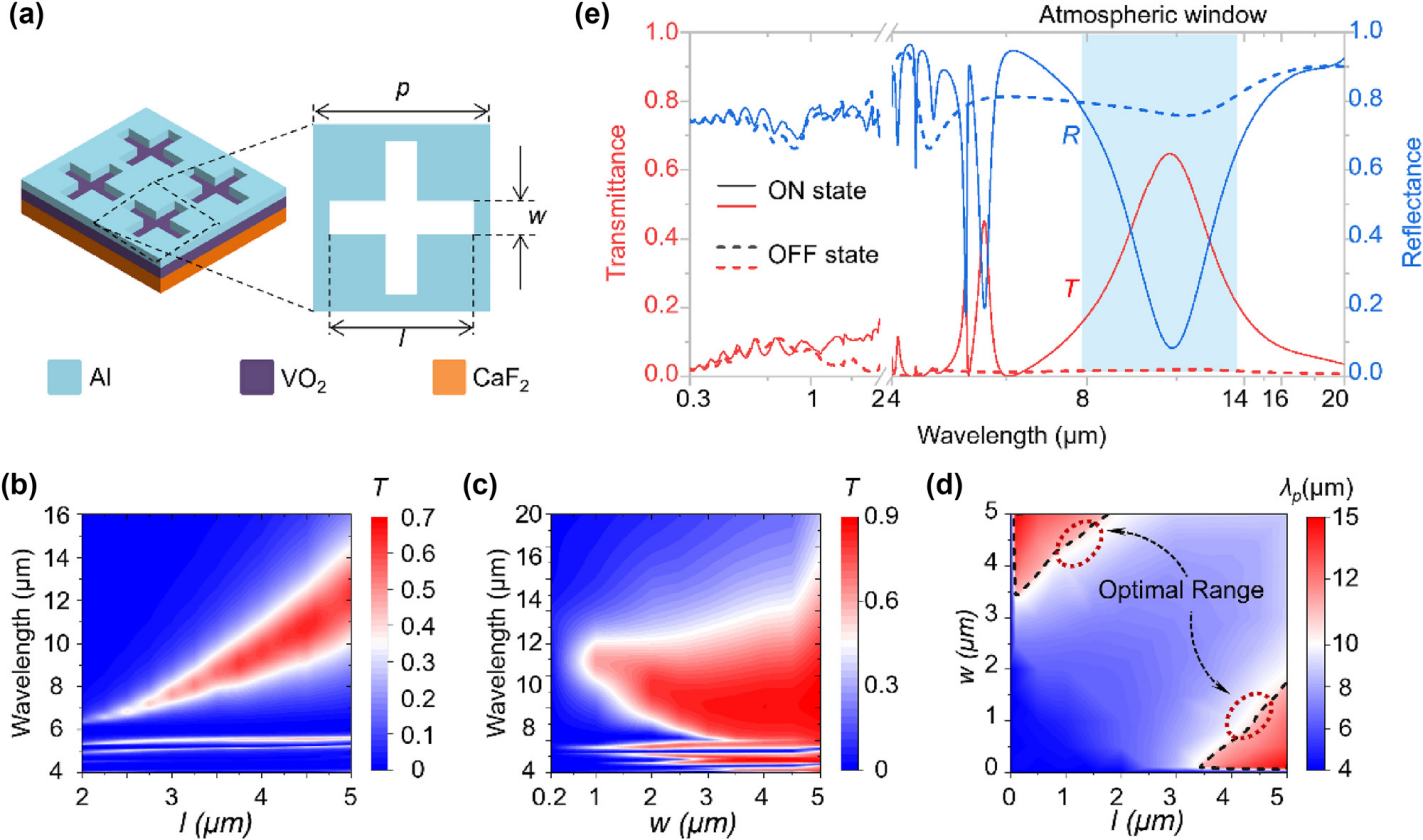
Device structure and the spectral response modulated by structure parameters. (a) Detailed configuration of the mid-infrared switch system, consisting of the CaF_2_ infrared-window layer, the VO_2_ phase-change layer, and the Al nanostructured metasurface. (b) Transmittance (red line) and reflectance (blue line) of the infrared switch with optimized feature size. The solid lines represent the ON state case and the dashed lines represent the OFF state. (c–d) Contour maps of the transmittance spectra as a function for (c) *l* and (d) *w,* respectively. (e) Transmittance peaks (*λ*_p_) under different (*l*, *w*) combinations. The dashed contour line highlights the transmittance peak at the wavelength of 10.6 μm.

Then, to find the optimal structural parameters satisfying the demands for scalable sizes and tunable spectral features, we simulate the temperature-dependent transmittance and reflectance spectra by COMSOL Multiphysics software (see details in Section 4) As shown in Figure 2a, the device comprises a CaF_2_ infrared window layer, a VO_2_ phase-change layer, and an Al metasurface featuring a cross-slot array arranged in a square lattice. In the simulation, the thicknesses of CaF_2_, VO_2_ and Al layers are set to 1 mm, 50 nm and 200 nm after considering the experimental availability and experience.

To investigate the influence of structure parameters on the transmission resonance, contour maps of the transmittance spectra as a function of *l* and *w* are obtained and given in Figure 2b–d. As shown in Figure 2b and c, the transmittance peak redshifts as l increases and blueshifts as w increases, which indicates that both the transmittance and the resonance wavelength can be tuned flexibly by engineering the cross-slot size. Figure 2d displays the extracted resonance wavelengths for various combinations of *l* and *w*, with the 10.64 μm isolines highlighted by dashed contours, as an indicator for transmittance in the atmosphere window. Due to the physical symmetry, the contour map is symmetrical concerning the line *l* = *w*. Consequently, the top-left half of Figure 2d is numerically identical to the bottom-right half. Given the intuitive structural definition in Figure 2a, we focus on the bottom-right isoline corresponding to 10.64 μm. Traditional research mainly focuses on the horizontal region with *w* of around 100 nm, where the transmittance resonance is highly sensitive to *w* but relatively insensitive to *l*. The transmittance resonance is effectively activated under this parameter, however, the fabrication requires expensive electron beam lithography, which typically limits the sample size to a few hundred micrometers. Herein, we find that enlarging *w* along the dashed contour can keep the transmittance at a relative high level. Compared to smaller feature sizes, increasing *w* to around 1 μm does not alter the position of the transmission peak but broadens the bandwidth of the transmission spectrum [31]. Meanwhile, the device maintains excellent spectral selectivity, and retains high overall transmittance efficiency, achieving balance between performance, fabrication simplicity and processing scalability to larger dimensions.

At this point, the pitch, width, and length of each cross-slot unit are set to 5.5 μm, 1 μm, and 4.5 μm, respectively. In the ON state shown in Figure 2e, a single transmittance peak emerges within the atmospheric window, facilitating the collection of infrared signals by the thermal photodetectors. For instance, at the wavelength of approximately 10.64 μm, the transmittance peak reaches 64.74 % in the ON state but drops to as low as 1 % in the OFF state. Correspondingly, the reflectance spectrum undergoes an opposite transition, rising from 10 % to 80 % as the device shifts from the ON state to the OFF state. This dynamic switching functionality is confined to the mid-infrared range, ensuring that the device operates in a normally-off mode across other wavelengths. The spectral selectivity inherent in the design, which allows for the transmission of mid-infrared light while effectively blocking other wavelengths, is invaluable for infrared windows. This feature significantly mitigates the risk of laser intrusion across multiple wavelengths, thereby enhancing the safety of devices under complex conditions.

**Figure 3:**
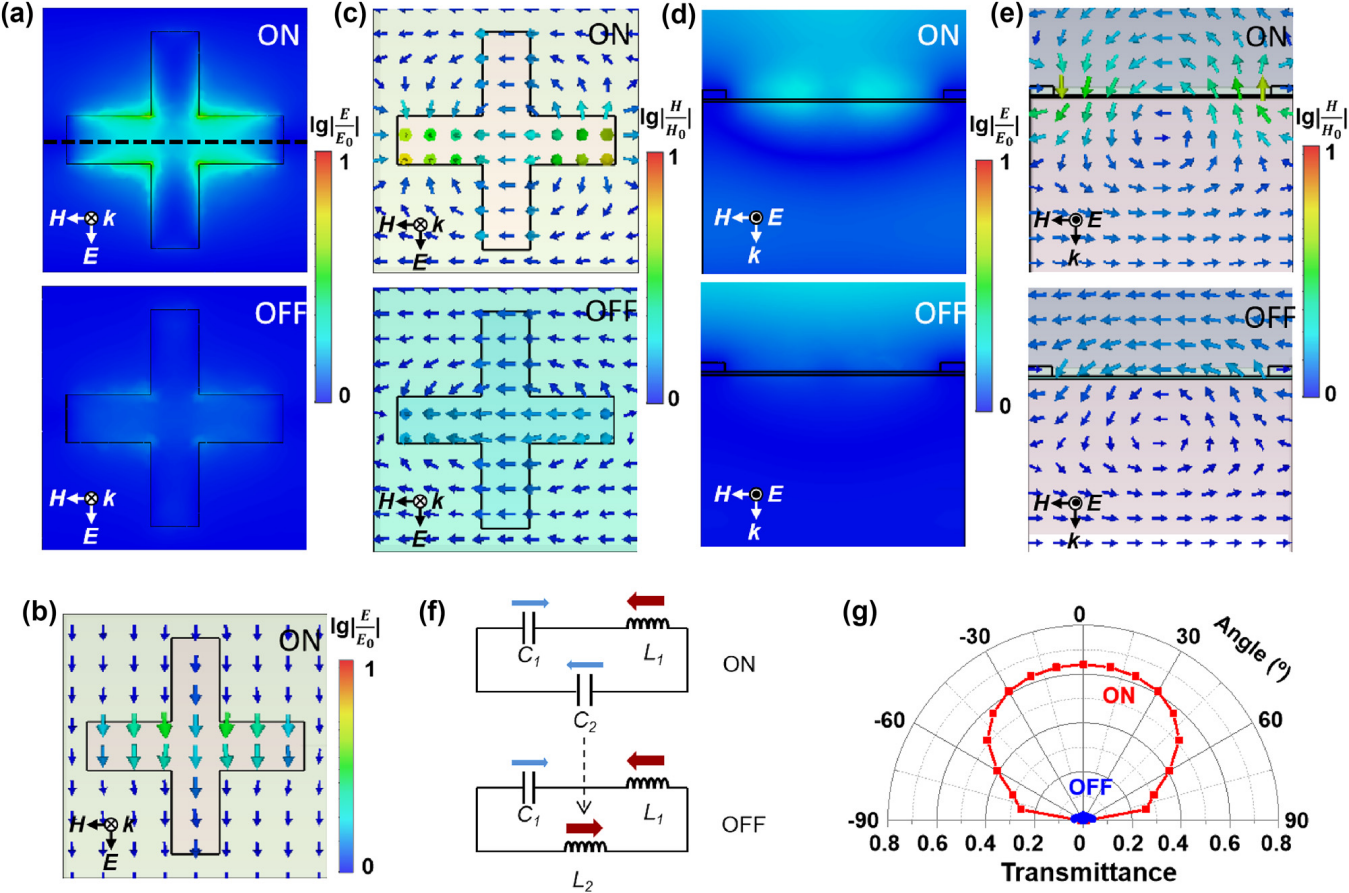
Physical mechanism of the adaptive mid-infrared switch. (a–e) Normalized electric and magnetic field distributions and vectors in the *x*–*y* (a, b, c) and *x*–*z* (d, e) planes, respectively. The magnitude is presented on a logarithmic scale for clarity. The top and bottom panels correspond to the ON and OFF states, respectively. (a) Electric field intensity in the *x*–*y* plane; (b) electric field vectors in the *x*–*y* plane; (c) magnetic field vectors in the *x*–*y* plane; (d) electric field intensity in the *x*–*z* plane; (e) magnetic field vectors in the *x*–*z* plane; (f) equivalent circuit model for the phase-change induced optical switch. (g) Azimuthal transmittance for the ON (red line) and OFF (blue line) states.

To explore the physical mechanism underlying the abnormal transmission near 10.64 μm and the phase transition-induced optical switching, we analyze the microscopic electromagnetic field distribution and equivalent circuit model. Figure 3a–e depict the magnitudes and directions of the normalized electromagnetic field in the horizontal *x*–*y* plane (i.e., top view) and the vertical *x*–*z* plane (i.e., side view). The top and bottom panels in Figure 3a and c–e correspond to the ON and OFF states, respectively. The intensity is normalized on a logarithmic scale to show the details clearly. In the ON state (top panel, Figure 3a), the light energy concentrated near the cross-slot indicates high transmission through the air cavity, which differs from the OFF state (bottom panel). It can be ascribed to the surface plasmon resonance, as evidenced by the electric vectors in Figure 3b, where a strong electric dipole forms across the air cross-slot. The physical origin is further confirmed by the contour map of magnetic vectors in Figure 3c. In both states, a magnetic dipole can be observed in the *x*–*y* plane, although the magnitude of the OFF state is much weaker than that of the ON state. Additionally, in the ON state, a significant magnetic vector component exists in the *x*–*z* plane, with a magnitude much greater than that of the *x*–*y* component. For a clearer understanding, the field distributions in the cross-section as indicated by the dash line in Figure 3a are given in Figure 3d and e. In the ON state (Figure 3d), the light energy penetrates the cross-slot and VO_2_ layer without impediment, indicating the successful coupling of the electromagnetic field to the air cavity. In contrast, in the OFF state, the transmission channel is closed due to the reflective nature of the metallic VO_2_ layer. As shown in Figure 3e, in the ON state, the surface plasmon-induced magnetic loop is clearly visible, with the strongest magnetic field along the sidewalls of the plasmonic cross-slots. In the OFF state, the magnetic loop shifts downward due to the significant phase delay of the metalized phase-change layer, and most of the magnetic field is blocked by the ultrathin metasurface.

The phase transition induced optical switching can also be elucidated through electronic circuit theory. As illustrated in Figure 3f, the optical metatronic circuit for the ON state can be represented as a lumped LC series circuit, where the plasmonic surface, the air cavity of the cross-slots, and the VO_2_ layer correspond to the inductor (L1), capacitor (C1) and another capacitor (C2), respectively. Based on the LC circuit resonance principle, when VO_2_ layer is insulating, the series capacitive impedance formed by C1 and C2 precisely counterbalances the inductive impedance of L1 in both magnitude and phase opposition, therefore establishing a minimal impedance matching state and locating in the optical transparent ON state. When the VO_2_ layer undergoes a phase transition from insulating to metallic states, the capacitor C2 transforms into an inductor L2 aligned parallel to L1 while maintaining other circuit parameters unchanged. This structural metamorphosis disrupts the original resonance equilibrium, thereby creating an impedance mismatch that corresponds to the optical opaque OFF state. The angular transmittance under ON and OFF states is investigated in Figure 3g. The ON state transmittance exceeds 0.6 within the range of ±30° and gradually decreases to 0.4 at an angle of 60°, exhibiting excellent angle-independence, while the transmittance keeps around 0 at all angles in the OFF state.

**Figure 4:**
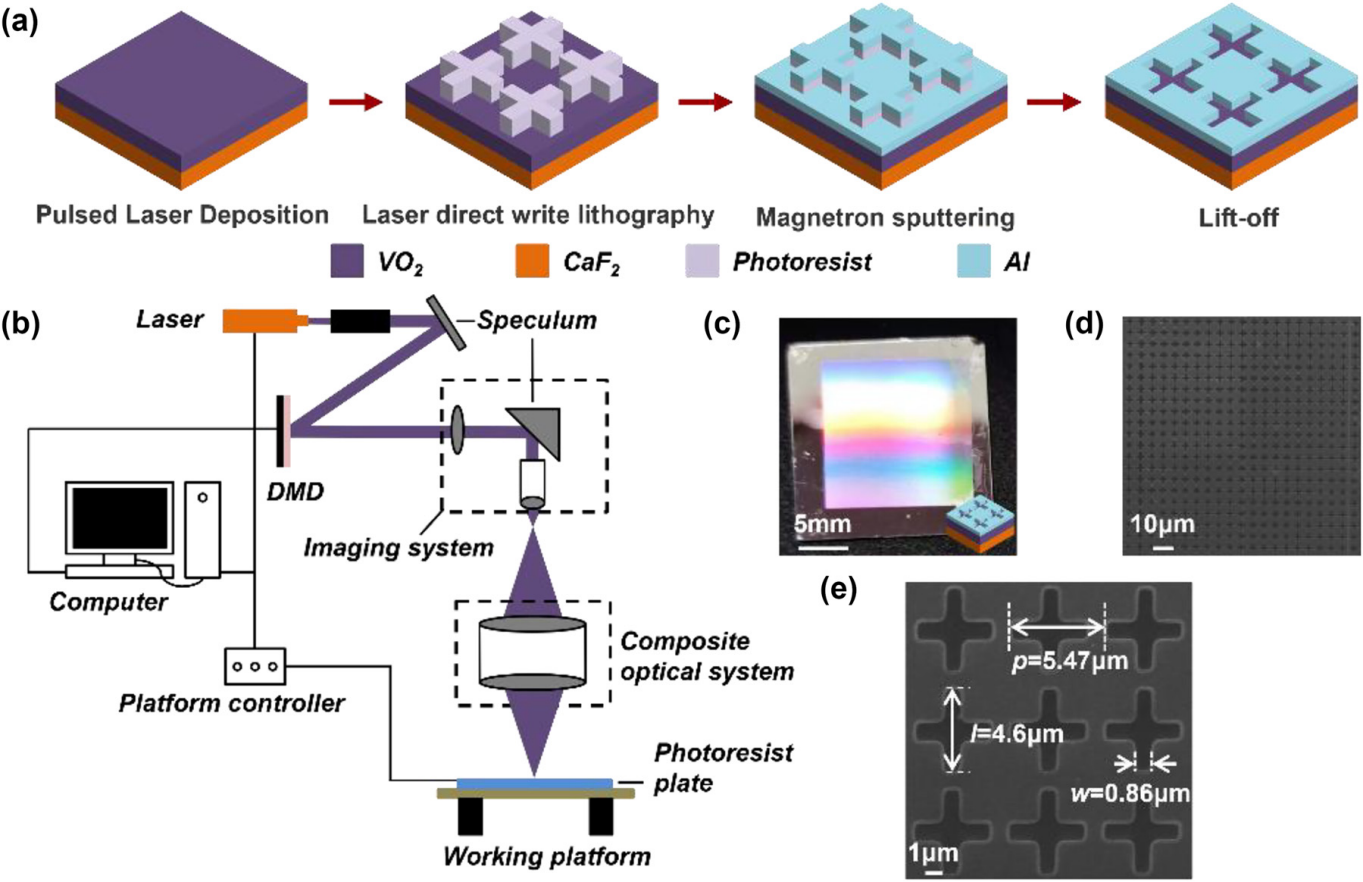
Device fabrication and morphology characterization. (a) Schematic diagram of the preparation process, which includes pulsed laser deposition, laser direct write lithography, magnetron sputtering, and lift-off steps. (b) Setup for laser direct write lithography. (c–d) Optical photograph (c) and microscopic SEM image (d) of the as-prepared device. (e) Enlarged SEM image with size labels.

Figure 4 illustrates the fabrication process and the microscopy images of the device. As shown in Figure 4a, the primary experimental steps include: (1) Growing VO_2_ thin film on the infrared CaF_2_ substrate using pulsed laser deposition (PLD); (2) Spinning photoresist coating and then generating the microscopic pattern complementary to the metasurface through laser direct write lithography (LDWL); (3) Depositing the Al layer via magnetron sputtering; and (4) Forming the final cross-slot metasurface using the lift-off process. Detailed experimental procedures can be found in the **Experimental Methods**. Figure 4b presents a schematic diagram of the homemade LDWL system, which consists of a beam expanding system, digital micromirror device (DMD), imaging system, composite optical system, and a micro-displacement system. The lithography process is primarily computer-controlled. Upon activation of the laser, the initial Gaussian-distributed light beam is expanded and homogenized using a beam expanding and collimating system, which increases the beam diameter and reduces its divergence angle. The light beam is then modulated by numerous micromirrors in DMD to form the target pattern, which is subsequently directed into the miniature imaging system to project the object pattern onto the substrate surface for exposure. Continuous refreshing of input patterns and movement of the sample stage enable the fabrication of large-area microstructures. Figure 4c gives the optical image of an as-prepared sample with an effective area of 15 × 15 mm^2^, displaying uniform visible dispersion, which indicates excellent fabrication quality on the macroscale. Enlarged SEM images are provided in Figure 4d and e, detailing the microstructure with *P* = 5.47 μm, *l* = 4.6 μm, and *w* = 0.86 μm.

**Figure 5:**
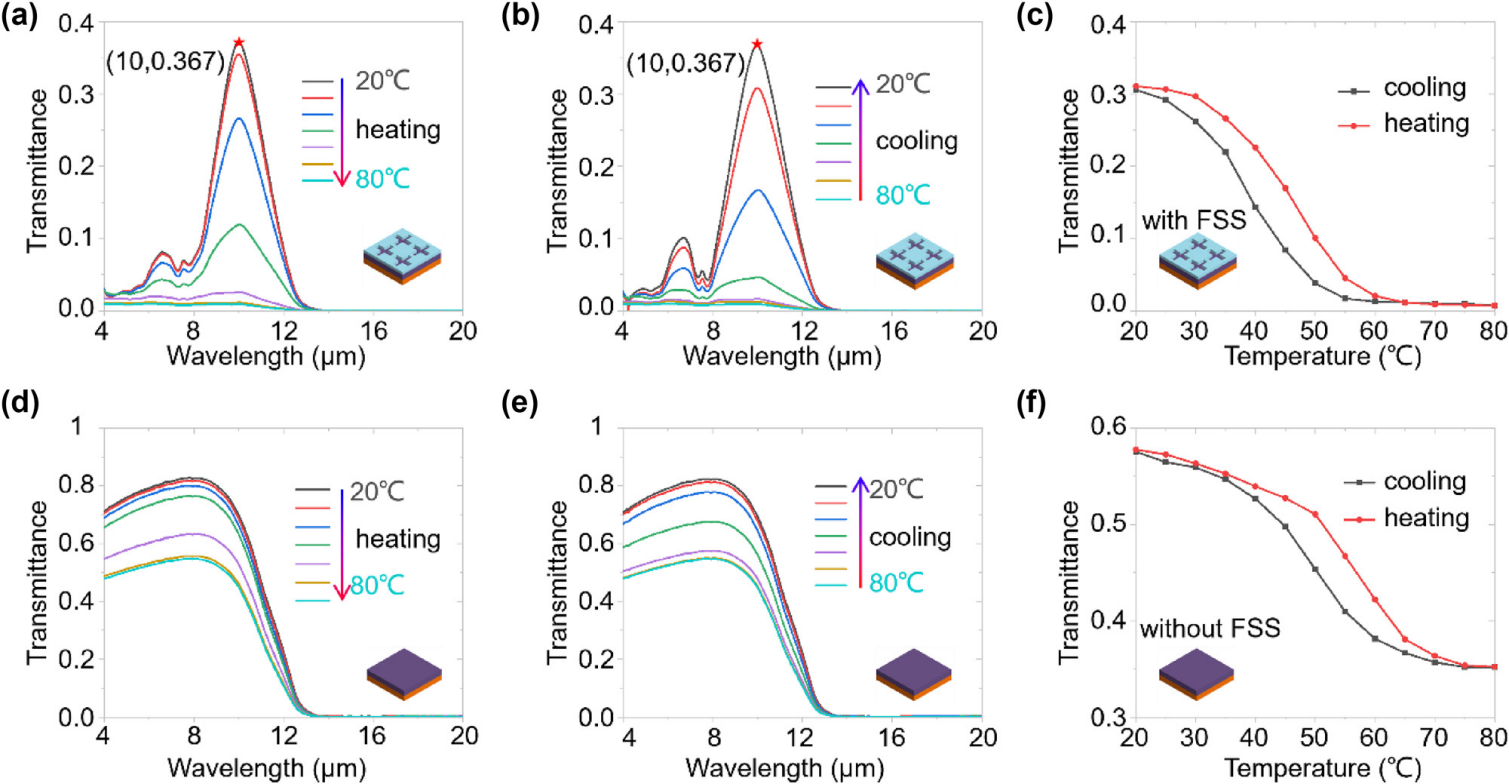
Measured transmittance spectra in the mid-infrared range during the heating and cooling process. (a–c) Are for the device with the cross-slot metasurface and (e–f) without the top metasurface. (a–b; d–e) Temperature-dependent transmittance spectra as the sample is heated up (a, d) and cooled down (b, e). (c, f) Hysteresis loop of the typical temperature-dependent transmittance at 10.64 μm.

Figure 5 gives the measured mid-infrared transmittance spectra during the heating and cooling processes, including both the metasurface device and its planar counterpart for direct comparison. It is worth noting that the phase transition of VO_2_ in our device is exclusively thermally triggered, which is in consistent with the actual thermal excitation via photothermal conversion under laser irradiation. As shown in Figure 5a, a transmittance peak appears at approximately 10.64 μm at room temperature, which is coincident well with the previous calculations. As the temperature increases from 20 °C to 30 °C, the peak decreases slightly; with further temperature increases, the transmittance dramatically decreases, approaching zero in a saturated closed state at ∼70 °C. The change in transmittance spectra during the cooling process is shown in Figure 5b. During cooling, the transmittance peak re-emerges at 50 °C and restores to the steady open state as the temperature gradually returns to room temperature. The reversible optical responses share the same steady states but follow different intermediate transition paths. As depicted in Figure 5c, the transmittance at a specific wavelength (e.g., 10.64 μm) during heating and cooling forms a reversible hysteresis loop with a narrow transition region.

In comparison to the metasurface device, the planar counterpart exhibits higher transmittance and excellent tunability. However, as shown in Figure 5d and e, the skin depth of metallic VO_2_ film in the whole mid-infrared region is higher than 50 nm, resulting in lack of ON/OFF switching capability for the planar counterpart, as well as the spectral selectivity. Furthermore, as shown in Figure 5f, during the heating process (red line), the rate of the transmittance decline per degree of temperature increase is much lower than that of the metasurface device. This difference is primarily due to the additional metallic top coating on the metasurface device, which enhances heating efficiency and improves the response time to external thermal effects from laser irradiation.

**Figure 6:**
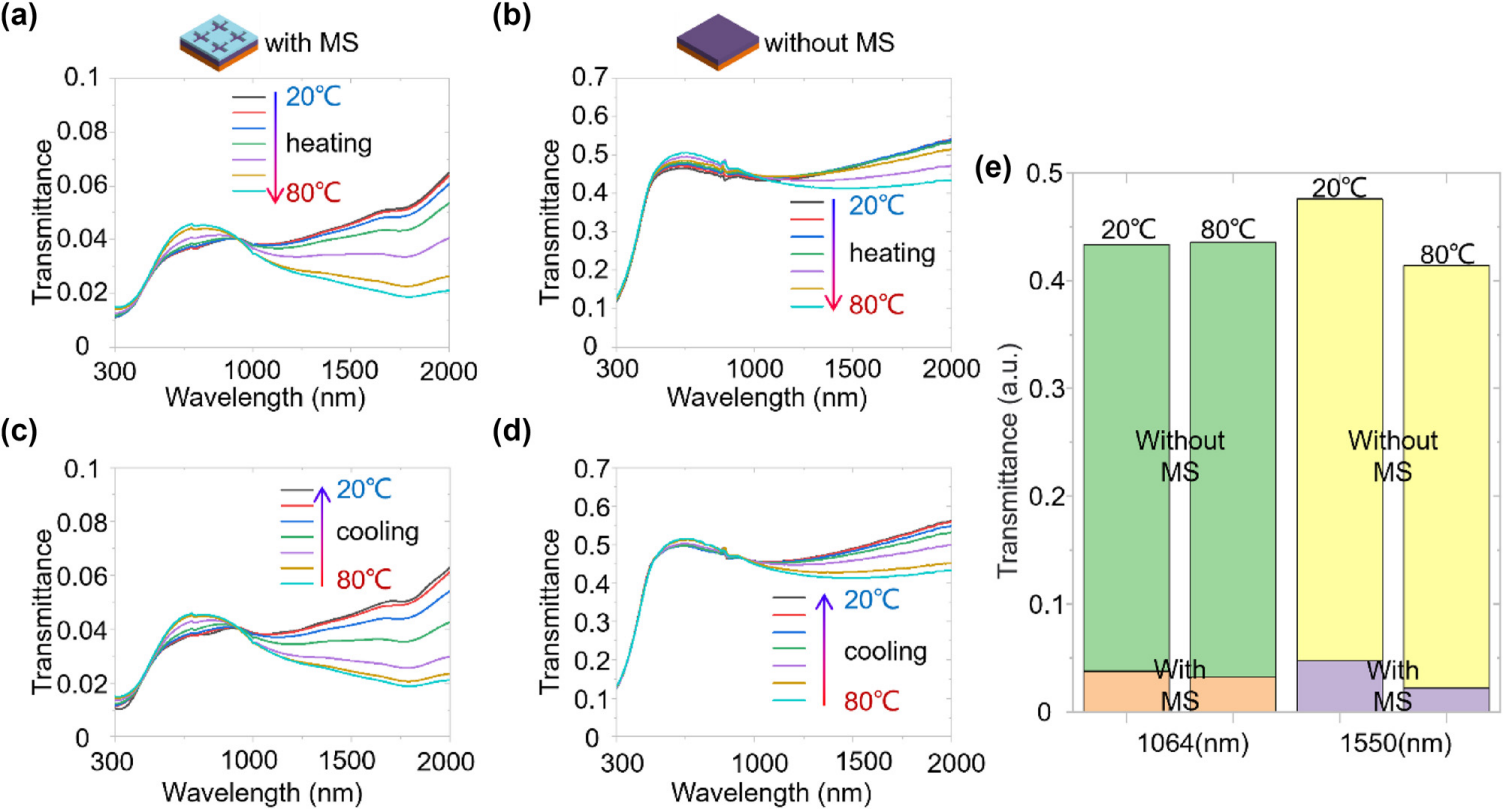
Measured UV-Vis-NIR transmittance spectra during the heating and cooling processes. (a–b) Spectra during the heating process. (c–d) Spectra during the cooling process. (e) Histogram comparing the transmittance of the device with and without the metasurface at specific temperatures and wavelengths.

While the device operates in the mid-infrared range, it is essential for other spectral ranges to remain in a normally off state, particularly for laser protection applications. To investigate this spectral selectivity, Figure 6 presents the transmittance spectra across the UV-Vis-NIR range for the device with and without the metasurface. The top panels (Figure 6a and b) correspond to the heating process, while the bottom panels (Figure 6c and d) correspond to the cooling process. Their comparison clearly shows that the transmittance of the device with the metasurface is approximately an order of magnitude lower than that of the planar counterpart, during both heating and cooling process. This demonstrates the significant advantage of the metasurface device in providing effective optical filtering across the ultraviolet to near-infrared range. As shown in Figure 6a, b, and e, at room temperature (i.e., 20 °C), the transmittance of the metasurface-based device is as low as 0.045 and 0.054 at 1,064 nm and 1,550 nm, respectively. Notably, these values decrease further to 0.040 and 0.026 as the temperature increases from 20 °C to 80 °C. In contrast, the planar counterpart exhibits much higher transmittance values, with 0.44 at 1,064 nm and 0.47 at 1,550 nm at 20 °C, which remain relatively stable at 1,064 nm and decrease slightly to 0.42 at 1,550 nm as the temperature rises to 80 °C. The transmittance of the metasurface device decreases with increasing temperature, showcasing its temperature-dependent spectral selectivity, unlike the planar counterpart whose transmittance remains relatively stable with temperature changes. During the cooling process, the transmittance spectra generally revert in the opposite manner observed during heating. As shown in Figure 6c and d, when the temperature decreases from 80 °C to 20 °C, the transmittance in the near-infrared (e.g., 1,064 nm and 1,550 nm) increases gradually in varying degrees.

## 3 Conclusions

In conclusion, we have successfully developed a dynamic and spectrally selective infrared switch comprising a cross-slot Al metasurface and VO_2_ layer that makes substantial strides in both functionality and manufacturability. This innovative device exhibits remarkable performance, efficiently transmitting mid-infrared light with a transmittance of 0.367 at 10.64 μm in the ON state, while transforming to a near-zero transmittance in the OFF state. Notably, it consistently demonstrates suppressed transmission across other wavelengths, particularly in the near-infrared region, underscoring its spectral selectivity. By scaling the minimum feature size of the metasurface array to approximately 1 μm, we have enhanced fabrication feasibility nearly tenfold compared to existing reports. This advancement not only simplifies the manufacturing process but also facilitates large-scale production, positioning our prototype as a promising candidate for practical applications. Ultimately, our findings contribute to the development of intelligent infrared windows and deepen our understanding of light–matter interactions in reconfigurable photothermal devices, paving the way for future innovations in this rapidly evolving field.

## 4 Methods

### 4.1 Sample preparation and optical measurements

The CaF_2_ (20 mm × 20 mm, polycrystalline) of 1 mm thick was used as a substrate, which was pre-processed by ultrasonic cleaning with ethyl alcohol, acetone, isopropanol, and de-mineralized water in sequence. A layer of 50 nm VO_2_ material was deposited on the CaF_2_ substrate by pulsed laser deposition (PLD, COMPEX PRO 205F) with a V_2_O_5_ ceramic target. The oxygen pressure for growth was 1.3 Pa, the temperature of substrate was 600 °C, and the deposition time was 45 min. We built a 200-nm aluminum FSS on top of the as-grown VO_2_ via the following steps. First, a 800-nm positive photoresist (SPR-955 0.9) was spin-coated at 3,000 R/min onto the VO_2_ film. A baking process followed the spin coating, and then the pattern was written using an laser direct writing system (MISCAN 200). After development in a diluted developer (NMD-3 2.38 %, volume ratio to water is 1:9), an array of cross-shape photoresist blocks were left on top of the VO_2_ film. It was then baked to solidify the shape of the cross. Later, A 200 nm Al layer was plated by magnetron sputtering (VTC-600-2HD), and the sample was soaked in an acetone bath at 60 °C for a few minutes to lift-off the photoresist blocks. The cross-section of samples was observed in SEM (Phenom-World BV, Eindhoven, the Netherlands). The reflection (*R*) and transmission (*T*) spectra came from the UV-Vis-NIR spectrophotometer (Lambda-950, PerkinElmer) with a wavelength range of 250–2,500 nm. The absorption spectra of the total system were derived from the conservation law of energy (i.e., *A* = 1 – *R* – *T*). The dynamic emissivity spectra of the prepared VO_2_ device were measured during the heating and cooling cycle by using a VERTEX 70 (Bruker) FTIR spectrometer with an A562 integrating sphere. The phase transition of VO_2_ was totally induced by external heating, with no electrical or optical triggering applied in this study.

### 4.2 Full-wave numerical simulations

The full-wave optical calculation was carried out by Comsol Multiphysics. The frequency-domain solver was employed to determine the electromagnetic field distribution within the multilayer system when subjected to the monochromatic plane-wave source. The wavelength parameter was swept incrementally by 1 nm. The absorptance was calculated by using $A={\int \;\int \;\int \;}_{v}\frac{1}{2}\omega \cdot \varepsilon^{\prime \prime }\left(\omega \right){\left|E\right|}^{2}dv$ , where *v* was the volume of active absorber layer, |*E*|^2^ was the normalized light intensity at the near field, *ε"* was the imaginary part of the material dielectric function of each layer. The calculated emissivity was set to be equal to the absorptivity according to Kirchhoff’s law. The complex refractive indexes of the CaF_2_, VO_2_, and Al material were derived from the experimental data [9], [32], [33], [34].
